# Vagus nerve stimulation even after injury ameliorates cisplatin-induced nephropathy via reducing macrophage infiltration

**DOI:** 10.1038/s41598-020-66295-0

**Published:** 2020-06-11

**Authors:** Rie Uni, Tsuyoshi Inoue, Yasuna Nakamura, Daichi Fukaya, Sho Hasegawa, Chia-Hsien Wu, Rie Fujii, Bongkod Surattichaiyakul, Wachirasek Peerapanyasut, Atsuko Ozeki, Nobuyoshi Akimitsu, Youichiro Wada, Masaomi Nangaku, Reiko Inagi

**Affiliations:** 1 Division of Nephrology and Endocrinology, The University of Tokyo Graduate, School of Medicine, Bunkyo-ku Tokyo, 113-8655 Japan; 2 Division of CKD, Pathophysiology, The University of Tokyo Graduate School of Medicine, Bunkyo-ku Tokyo, 113-8655 Japan; 3 Mahidol University, Nakhonsawan Campus, Nakhonsawan, 60130 Thailand; 4 Isotope Science Center, The University of Tokyo, Bunkyo-ku Tokyo, 113-0032 Japan

**Keywords:** Nephrology, Acute kidney injury, Toxin-induced nephropathy

## Abstract

The efficacy of prior activation of an anti-inflammatory pathway called the cholinergic anti-inflammatory pathway (CAP) through vagus nerve stimulation (VNS) has been reported in renal ischemia-reperfusion injury models. However, there have been no reports that have demonstrated the effectiveness of VNS after injury. We investigated the renoprotective effect of VNS in a cisplatin-induced nephropathy model. C57BL/6 mice were injected with cisplatin, and VNS was conducted 24 hours later. Kidney function, histology, and a kidney injury marker (Kim-1) were evaluated 72 hours after cisplatin administration. To further explore the role of the spleen and splenic macrophages, key players in the CAP, splenectomy, and adoptive transfer of macrophages treated with the selective α7 nicotinic acetylcholine receptor agonist GTS-21 were conducted. VNS treatment significantly suppressed cisplatin-induced kidney injury. This effect was abolished by splenectomy, while adoptive transfer of GTS-21-treated macrophages improved renal outcomes. VNS also reduced the expression of cytokines and chemokines, including CCL2, which is a potent chemokine attracting monocytes/macrophages, accompanied by a decline in the number of infiltrating macrophages. Taken together, stimulation of the CAP protected the kidney even after injury in a cisplatin-induced nephropathy model. Considering the feasibility and anti-inflammatory effects of VNS, the findings suggest that VNS may be a promising therapeutic tool for acute kidney injury.

## Introduction

Despite the advancements in modern medical technology, acute kidney injury (AKI) is still one of the major comorbidities in hospital settings. It is estimated that AKI occurs in approximately 15% of hospitalized patients and 60% of critically ill patients^1^, and morbidity and mortality rates remain high^2,3^. In addition, AKI is a risk factor for chronic kidney disease (CKD) and end-stage renal disease (ESRD)^4^. Therefore, prevention of AKI development and progression to CKD is essential. Inflammation plays an important role in the pathogenesis of AKI^5^. Moreover, chronic inflammation contributes to the progression of CKD. Therefore, suppression of inflammation plays a potential role in treating kidney injury.

Recently, a new anti-inflammatory pathway called the cholinergic anti-inflammatory pathway (CAP) has been discovered^6^. The CAP consists of both afferent and efferent arms, and both afferent and efferent vagus nerves play important roles. The afferent vagus nerve conducts inflammatory information from the peripheral organs to the central nervous system. In the brainstem, the afferent vagus nerve activates the C1 neurons, which make a major contribution to the central regulation of autonomic function^7^, and further stimulate the efferent vagus nerve^8^.

Previously, Inoue and Abe *et al*. reported that vagus nerve stimulation (VNS) protected the kidney from ischemia-reperfusion injury (IRI) through activation of the CAP^9^. Although there are many kinds of inflammatory cells such as B cells, T cells, and dendritic cells in the spleen, the anti-inflammatory effect of CAP stimulation is delivered through activation of α7 nicotinic acetylcholine receptor (α7nAChR) on splenic macrophages^10^. Considering its anti-inflammatory effect, VNS is a potent tool for treating inflammatory disorders such as sepsis, lung injury, rheumatoid arthritis, inflammatory bowel disease, and diabetes^11^. VNS was approved by the Food and Drug Administration in 1997 for the treatment of refractory partial-onset epilepsy^12^ and in 2005 for chronic or recurring depression^13^. The VNS device is a battery-powered apparatus akin to a cardiac pacemaker. In most models, stimulating electrical leads are surgically implanted in the carotid sheath around the left vagus nerve. Its safety is proven in that more than 100,000 VNS devices have been implanted in over 75,000 patients worldwide^14^. Moreover, noninvasive transcutaneous VNS devices have also been developed^15,16^, wherein the vagus nerve is stimulated via the auricular branch of the nerve by small, earphone-like electrodes. A pilot study demonstrated that noninvasive VNS downregulated inflammatory cytokine release in healthy subjects^16^. These results reinforce the fact that VNS is safe and readily applicable in hospital settings. Given the high prevalence of AKI and the difficulty of identifying patients who will develop AKI, it would be more beneficial if VNS has therapeutic effect, even after injury.

Cisplatin is a major tumoricidal drug that has long been used for the treatment of a number of cancers. Cisplatin induces AKI, an important dose-limiting toxicity that frequently leads to cessation of therapy^17^. In addition, cisplatin is used to produce one of the common animal AKI models.

In this study, we hypothesized that VNS is renoprotective after the development of AKI, and we investigated this using a cisplatin-induced nephropathy model.

## Results

### Cisplatin causes tubular damage 24 hours after its administration

Although the plasma creatinine and blood urea nitrogen (BUN) levels were not significantly elevated 24 hours after cisplatin injection (Supplementary Figure 1a,b), histology demonstrated the early stage of tubular injury characterized by degenerative changes of proximal tubules (Supplementary Fig. 1c,d). The expression of kidney injury molecule-1 (Kim-1) and neutrophil gelatinase-associated lipocalin (Ngal) mRNA were also elevated in the cisplatin group compared to the control group (Supplementary Fig. 1e,f).

### VNS after cisplatin injection protected kidney injury

**Figure 1:**
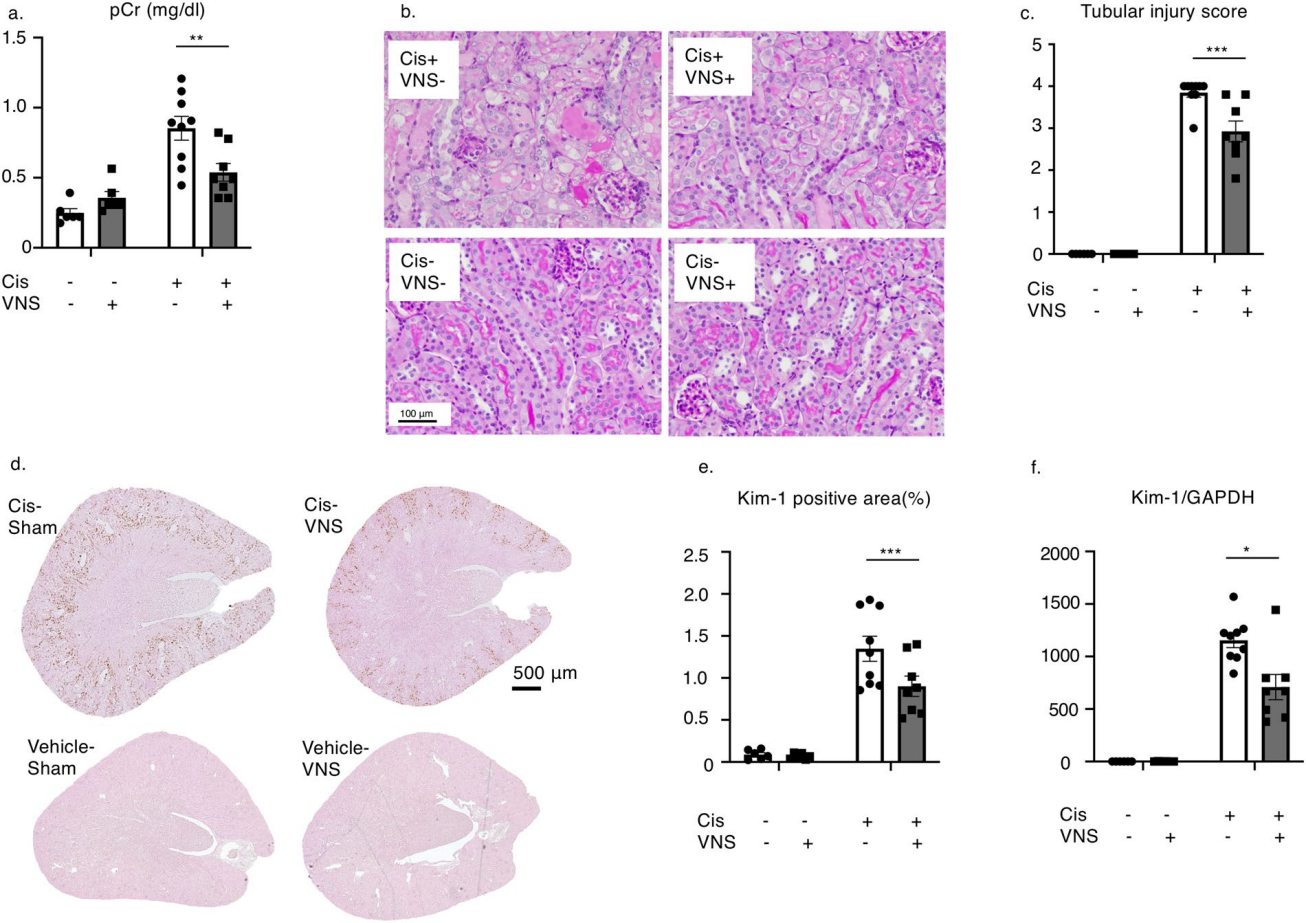
Effectiveness of VNS after cisplatin injection. VNS is applied 24 hours after intraperitoneal single injection of cisplatin (25 mg/kg). (**a**) Plasma creatinine is significantly decreased in the VNS-treated group (plasma creatinine: 0.85 ± 0.09 and 0.53 ± 0.06 mg/dl for Cis-sham and Cis-VNS, 0.25 ± 0.03 and 0.36 ± 0.04 mg/dl for vehicle-sham and vehicle-VNS, respectively; n = 8 or 9; *P* = 0.0031). (**b**,**c**) Representative pictures of PAS staining and tubular injury score. VNS attenuates the degree of tubular injury (tubular injury score: 3.84 ± 0.11 and 2.93 ± 0.25 for Cis-sham and Cis-VNS, 0.0 ± 0.0 and 0.0 ± 0.0 for vehicle-sham and vehicle-VNS, respectively; *P* = 0.0002). (**d–f**) The expression of tubular injury marker, Kim-1, is downregulated by VNS. The area fraction of the Kim-1-positive area is analyzed using Image-J software (Kim-1-positive area: 1.35 ± 0.15 and 0.90 ± 0.12% for Cis-sham and Cis-VNS, 0.09 ± 0.02 and 0.07 ± 0.01% for vehicle-sham and vehicle-VNS, respectively; n = 8 or 9; *P* = 0.0133). Kim-1 expression in the whole kidney is also reduced by VNS (Cis-sham 1154.3 ± 70.0 and Cis-VNS 708.8 ± 119.5-fold change compared with vehicle-sham; *P* = 0.0005). Data are expressed as mean ± SEM. Scale bar, 100 μm. *P < 0.05, **P < 0.01, ***P < 0.001 (two-way analysis of variance followed by Sidak post-hoc test. VNS, vagus nerve stimulation; Cis, cisplatin; PAS, periodic acid-Schiff; SEM, standard error of the mean.

Here, we applied VNS 24 hours after cisplatin injection and evaluated kidney functions in 72 hours. At 72 hours after cisplatin administration, plasma creatinine levels were increased, but decreased significantly after VNS (Fig. 1a). Histology showed that VNS significantly improved cisplatin-induced tubular injury, characterized by a decreased number of apoptotic or necrotic tubular epithelial cells and tubular detachments (Fig. 1b,c). Increased Kim-1 expression induced by cisplatin administration was significantly decreased by VNS treatment as seen on immunohistochemistry of the kidney (Fig. 1d,e, Supplementary Fig. 2). This was further confirmed by the change in Kim-1 mRNA expression in the kidney by real-time PCR (Fig. 1f).

### Splenectomy abolished renoprotective effect of VNS

**Figure 2:**
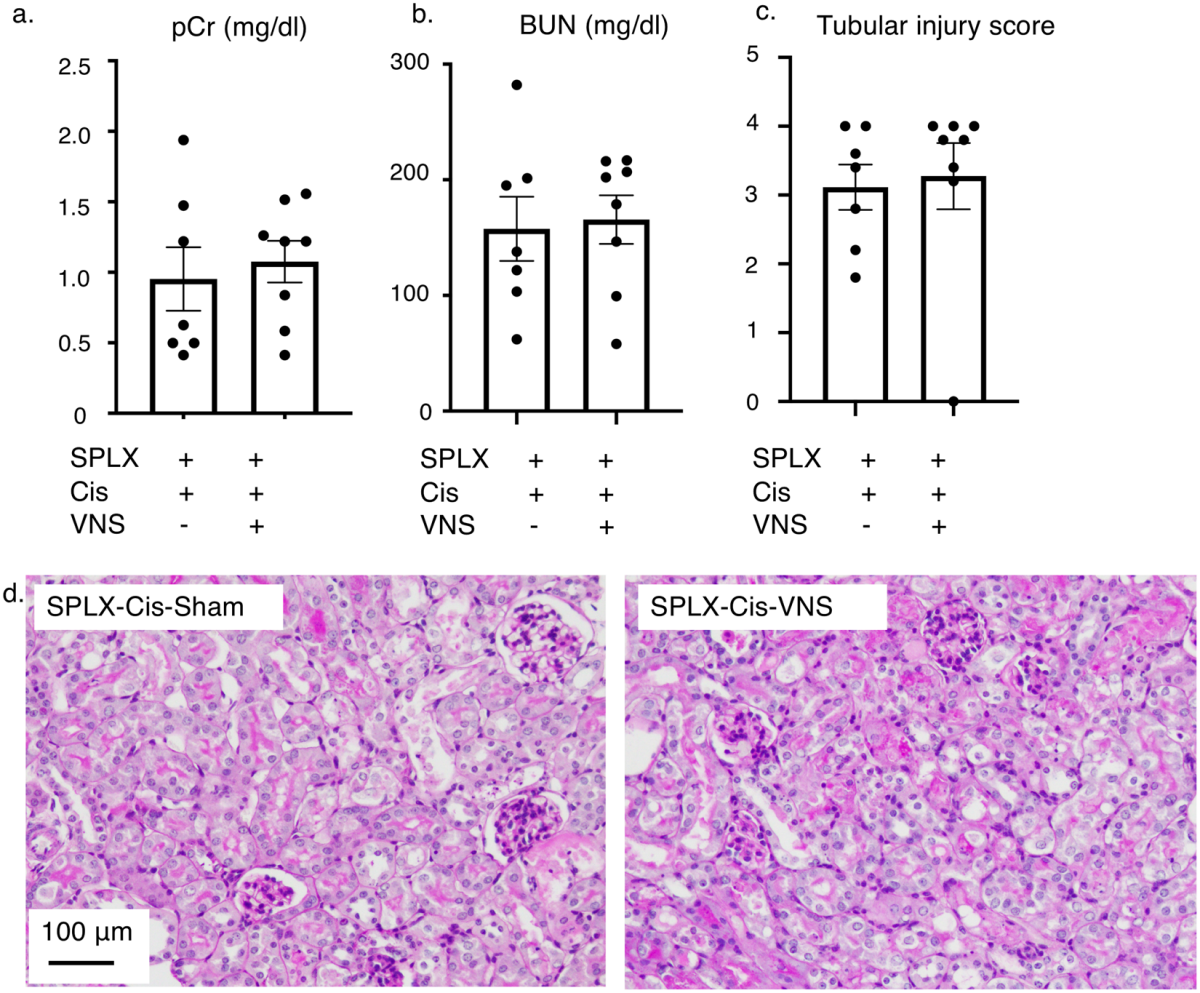
Splenectomy before cisplatin injection abolishes the renoprotective effect of VNS. (**a–c**) Plasma creatinine, BUN, and histology score do not differ between the two groups (plasma creatinine: 0.95 ± 0.22 and 1.08 ± 0.15 mg/dl; BUN: 157.7 ± 27.8 and 165.6 ± 21.0 mg/dl; histology score: 3.11 ± 0.30 and 3.28 ± 0.49 mg/dl; for SPLX-Cis-sham and SPLX-Cis-VNS, respectively; n = 7 or 8). (d) Representative pictures of PAS-staining. Data are expressed as mean ± SEM and analyzed using a Student’s t-test. Scale bar, 100 μm. BUN, blood urea nitrogen; VNS, vagus nerve stimulation; PAS, periodic acid-Schiff; SEM, standard error of the mean; SPLX, splenectomy.

Since the spleen is one of the essential components of the CAP and splenectomy completely eliminates the renoprotective effect of VNS in ischemia-reperfusion injury models^9^, we conducted splenectomies 5 days before cisplatin injection to evaluate the contribution of the spleen to the renoprotective effect of VNS in the cisplatin-induced nephropathy model. Renoprotection was not observed in the splenectomized mice. There was no statistical difference between the two groups in terms of plasma creatinine and BUN levels (Fig. 2a,b). We also evaluated the extent of the tubular injury, which showed that VNS did not attenuate the tubular damage in splenectomized mice (Fig. 2c,d).

### Adoptive transfer of GTS-21-treated macrophages improved renal outcome in the cisplatin-induced nephropathy model

**Figure 3:**
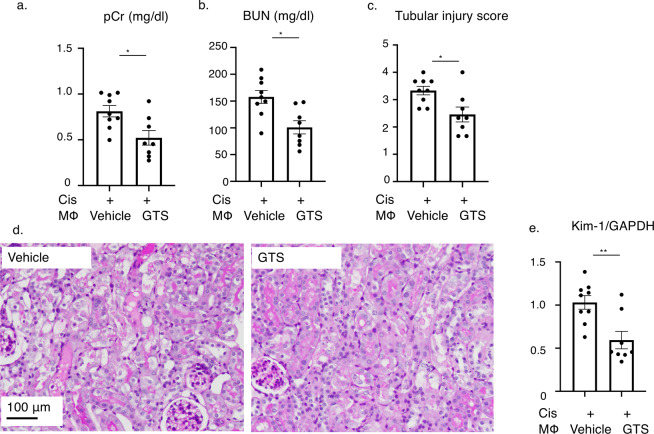
Adoptive transfer of α7nAChR agonist GTS-21-treated macrophages improved renal outcome. (**a–c**) Plasma creatinine, BUN, and histology score are significantly reduced by GTS-treated macrophage transfer (plasma creatinine: 0.81 ± 0.06 and 0.51 ± 0.07 mg/dl, *P* = 0.0105; BUN: 157.8 ± 11.4 and 101.0 ± 11.6 mg/dl, *P* = 0.0051; histology score: 3.33 ± 0.15 and 2.46 ± 0.26, *P* = 0.0122; for Cis-vehicle-treated macrophage and Cis-GTS-treated macrophage, respectively; n = 8 or 9). (**d**) Representative pictures of PAS-staining. (**e**) The expression level of Kim-1 mRNA in the whole kidney was also decreased by GTS-21-treated macrophage transfer (relative expression of Kim-1: 1.031 ± 0.07 and 0.59 ± 0.09, *P* = 0.0034; for Cis-vehicle-treated macrophage and Cis-GTS-treated macrophage, respectively; n = 8 or 9). Data are expressed as mean ± SEM. Scale bar, 100 μm. *P < 0.05, **P < 0.01, ***P < 0.001 (two-way analysis of variance followed by the Sidak post-hoc test). BUN, blood urea nitrogen; VNS, vagus nerve stimulation; Cis, cisplatin; SEM, standard error of the mean; GTS, GTS-21; PAS, periodic acid-Schiff.

Though many kinds of inflammatory cells, including B cells, T cells and dendritic cells, exist in the spleen, the anti-inflammatory effect of CAP stimulation is delivered through activation of the α7nAChR on splenic macrophages^10^. We conducted adoptive transfer of splenic macrophages (1.0×10^5 cells) either treated with the selective α7nAChR agonist GTS-21, or vehicle 24 hours after cisplatin injection. F4/80+ macrophages were collected using the magnetic cell separation method (MACS) and incubated with vehicle or GTS-21 for 1 hour, and then intravenously injected to recipient mice. Adoptive transfer of macrophages itself did not affect the kidney function of healthy control mice (data not shown), but at 72 hours after cisplatin injection, previously increased plasma creatinine and BUN levels were significantly decreased in the mice that had received the GTS-21-treated macrophages after disease induction (Fig. 3a,b). Tubular injury was also ameliorated by GTS-21-treated macrophage transfer (Fig. 3c,d). The expression level of Kim-1 in the kidney was also decreased significantly in the GTS-21-treated macrophages-injected group (Fig. 3e).

### Inflammatory cytokines are upregulated in the cisplatin-induced nephropathy model and reduced by VNS

**Figure 4:**
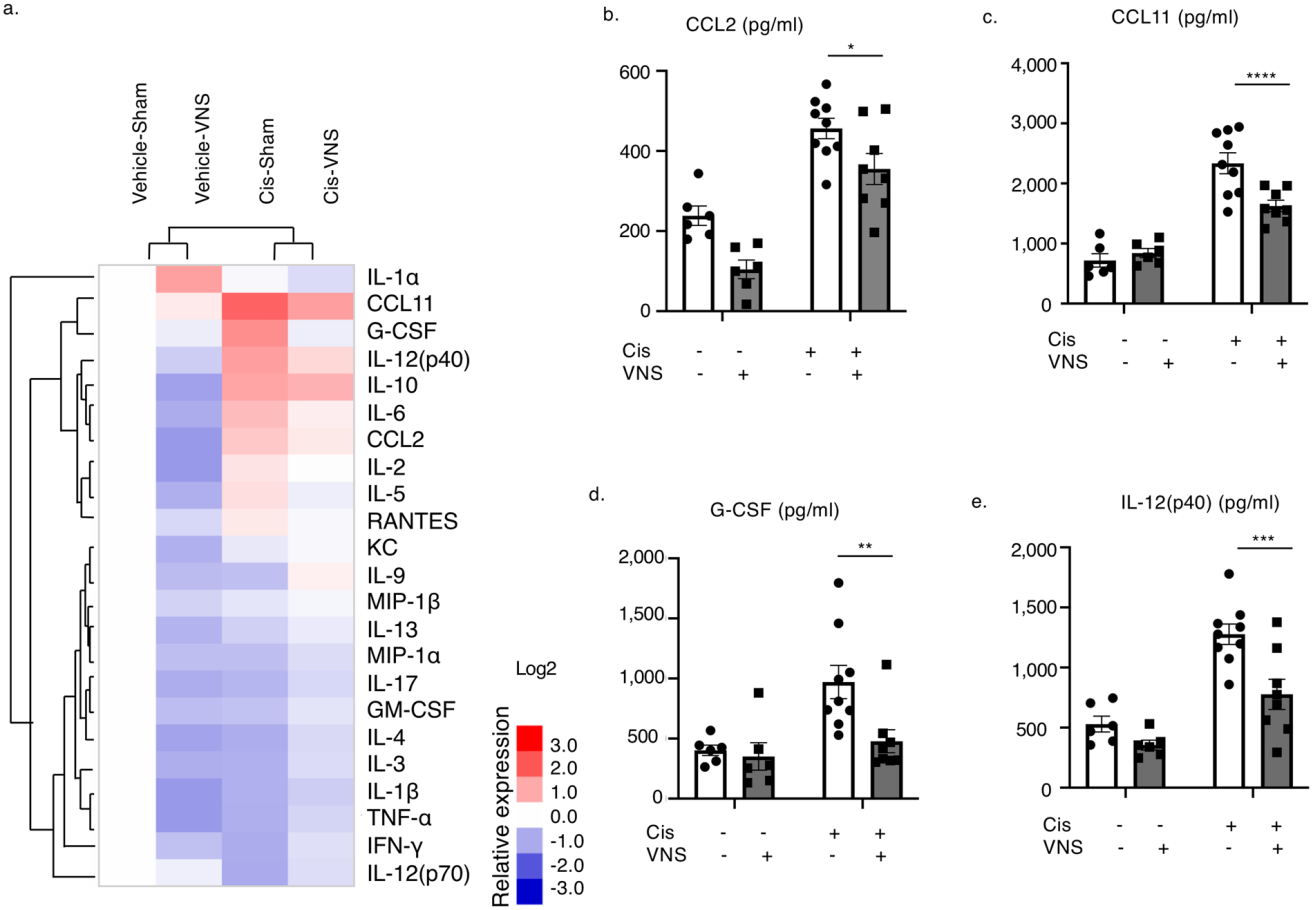
VNS improves systemic inflammation and decreased expression level of cytokines and chemokines in plasma. (**a**) VNS alone downregulates the cytokines evaluated. It also suppresses most of the cytokines upregulated by cisplatin injection (raw data in Supplementary Fig. 1). Clustering is performed to generate a heatmap using clustering software (n = 6–9). (**b**) CCL2 (CCL2: 368.9 ± 34.2 and 282.0 ± 36.6 pg/ml for Cis-sham and Cis-VNS, respectively; n = 6–9; *P* = 0.0325). (**c**) CCL11 (CCL11: 2565.5 ± 210.3 and 1559.1 ± 110.3 pg/ml for Cis-sham and Cis-VNS, respectively; n = 6–9; *P* < 0.0001). (**d**) G-CSF (G-CSF: 1010.4 ± 147.5 and 355.4 ± 66.4 pg/ml for Cis-sham and Cis-VNS, respectively; n = 6–9; *P* = 0.0002). (**e**) IL-12(p40) (IL-12(p40): 1277.4 ± 85.2 and 777.2 ± 125.9 pg/ml for Cis-sham and Cis-VNS, respectively; n = 6–9; *P* = 0.0008). Data are expressed as mean ± SEM. *P < 0.05, **P < 0.01, ***P < 0.001 (two-way analysis of variance followed by the Sidak post-hoc test). VNS, vagus nerve stimulation; Cis, cisplatin; SEM, standard error of the mean.

Next, in order to investigate the effect of CAP activation on systemic inflammation, we evaluated the expression of various cytokines in plasma and created a heatmap using clustering analysis (Fig. 4a; raw data in Supplementary Table 1).

Among 23 cytokines tested, the levels of four cytokines—CCL2, CCL11 (Eotaxin), G-CSF, and IL-12(p40)—were significantly reduced by VNS in cisplatin-induced nephropathy mice (Fig. 4b–e).

### Several cytokines in the kidney are elevated in the cisplatin-induced nephropathy model and downregulated by VNS

**Figure 5:**
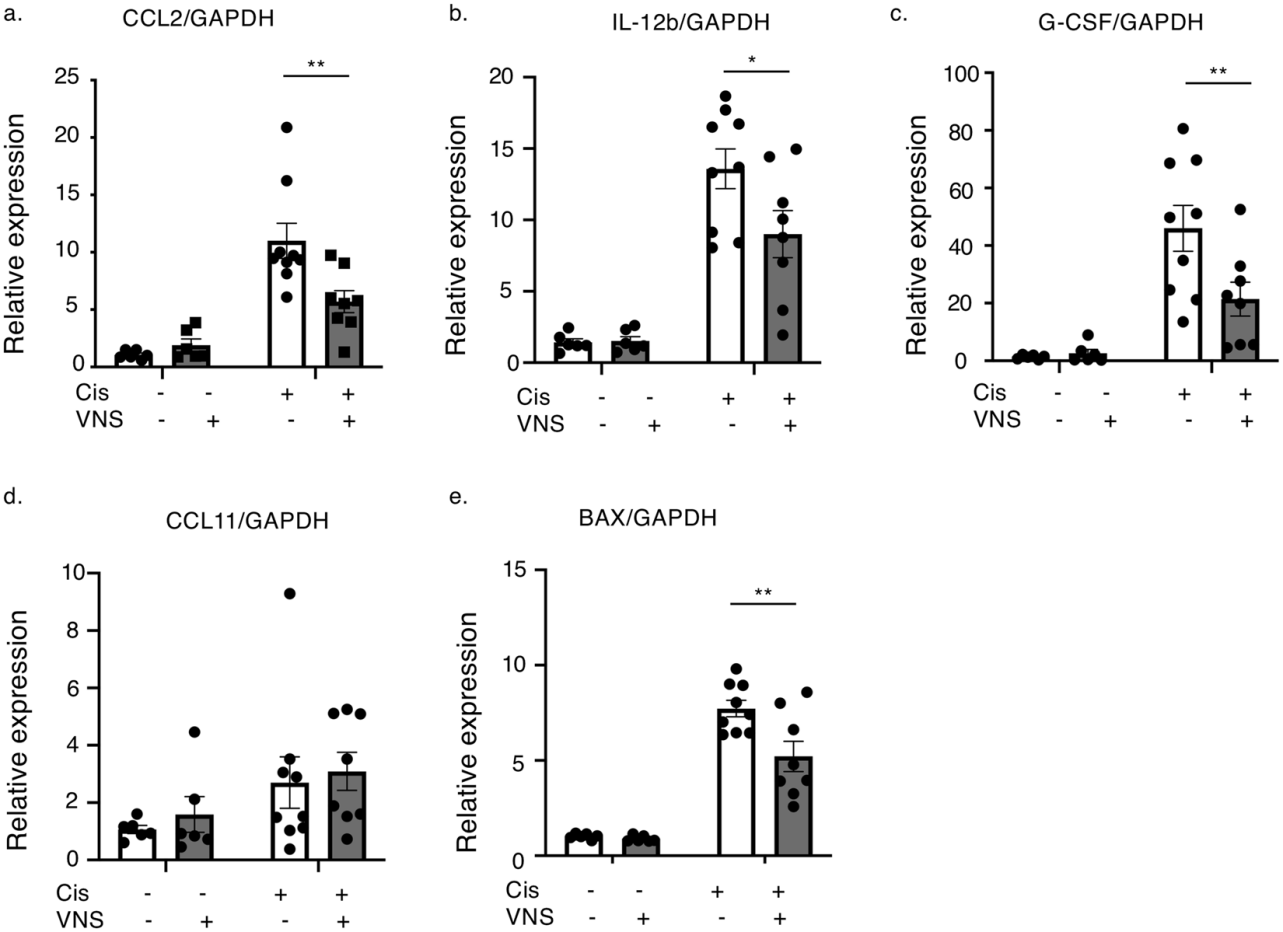
VNS reduces the expression of several inflammatory cytokines and chemokines in the kidney. (**a**) CCL2 expression (CCL2: 11.0 ± 1.53 and 5.70 ± 0.96-fold change compared with vehicle-sham for Cis-sham and Cis-VNS, respectively; n = 6–9; *P* = 0.0026). (**b**) IL-12b expression (IL-12b: 13.6 ± 1.34 and 9.0 ± 1.66-fold change compared with vehicle-sham for Cis-sham and Cis-VNS, respectively; n = 6–9; *P* = 0.0226). (**c**) G-CSF expression (G-CSF: 46.0 ± 7.96 and 21.4 ± 5.86-fold change compared with vehicle-sham for Cis-sham and Cis-VNS, respectively; n = 6–9; *P* = 0.0089). (**d**) CCL11 expression (CCL11: 13.6 ± 1.34 and 9.0 ± 1.66-fold change compared with vehicle-sham for Cis-sham and Cis-VNS, respectively; n = 6–9; *P* = 0.0226). (**e**) BAX expression (BAX: 7.7 ± 0.43 and 5.2 ± 0.79-fold change compared with vehicle-sham for Cis-sham and Cis-VNS, respectively; n = 6–9; P = 0.0022). Data are expressed as mean ± SEM. *P < 0.05, **P < 0.01, ***P < 0.001 (two-way analysis of variance followed by the Sidak post-hoc test). VNS, vagus nerve stimulation; Cis, cisplatin; SEM, standard error of the mean.

Real-time PCR showed a significant decline in the expression levels of CCL2, IL-12b, and G-CSF in the mice treated with cisplatin followed by VNS (Fig. 5a–d). Since cisplatin-induced nephropathy is characterized by mitochondrial damage of tubular cells and upregulated expression of BCL2-associated X protein (BAX), which is one of the major proteins inducing mitochondrial apoptosis by permeabilizing its membrane^18,19^, we evaluated the expression of BAX mRNA. As shown in Fig. 5e, cisplatin-induced expression of BAX was also suppressed by VNS.

### VNS reduces F4/80* +* macrophage infiltration into the injured kidney

**Figure 6:**
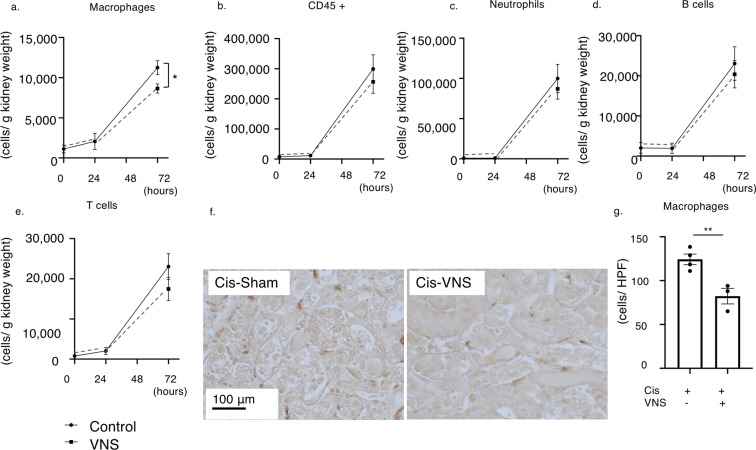
VNS significantly reduces the number of infiltrating macrophages in the kidney. (**a–e**) The number of infiltrating leukocytes is evaluated by flow cytometry analysis. (**a**) There is no significant difference 24 hours after cisplatin injection, but VNS significantly decreases macrophage infiltration 72 hours later (the number of infiltrating macrophages: 11261.1 ± 865.3 and 8660.8 ± 561.6 cells/ g kidney weight for Cis-sham and Cis-VNS, respectively; n = 4 per group; *P* = 0.0453). (**f**) Representative pictures of F4/80 staining of kidney sections. (**g**) The number of macrophages is counted manually using immunohistochemical staining of F4/80 positive cells. (124.3 ± 6.0 and 82.3 ± 8.8 cells/HPF for Cis-sham and Cis-VNS, respectively; n = 3 or 4; *P* = 0.0095). Data are expressed as mean ± SEM and analyzed using a Student’s t test. *P < 0.05. VNS, vagus nerve stimulation; Cis, cisplatin; HPF, high power field.

Next, as macrophages have been proven to be another key player in the CAP^10^ and CCL2, one of the most potent chemokines promoting monocyte and macrophage chemotaxis, was downregulated both in the kidney and blood, we hypothesized that CAP activation prohibits macrophage migration to the injured kidneys. Therefore, we evaluated the macrophage infiltration of the kidney in the VNS-treated or sham-treated mice after cisplatin administration by flow cytometry analysis and immunohistochemical staining of F4/80 positive macrophages. Flow cytometry analysis showed that, at 24 hours after cisplatin injection, the number of CD45-positive leukocytes, including macrophages and T cells, was slightly elevated, and the number of the cells in each leukocyte fraction was further increased 72 hours after cisplatin administration. In contrast, VNS significantly reduced the infiltration of macrophages by cisplatin (Fig. 6a–e). Immunohistochemical staining of F4/80-positive macrophages also demonstrated that the number of infiltrated macrophages in the kidney was also significantly decreased by VNS at 72 hours after cisplatin injection (Fig. 6f,g).

## Discussion

To the best of our knowledge, this is the first study to demonstrate the effectiveness of VNS after kidney injury. Regardless of the innovations in medicine, supportive and preventative methods such as hydration or avoidance of renal toxic drugs remain the main treatment options for AKI. No safe and effective therapeutic modalities for AKI have been established to date.

Formerly, Inoue and Abe *et al*. proved the efficacy of VNS before injury in an IRI model. Although the precise mechanism underlying the pathogenesis of AKI in toxin-induced nephropathy models and IRI models is unknown, inflammation after initial insult is the common pathway leading to the development of kidney injury^5^. Thus, we hypothesized that VNS would be universally effective for various AKI models. More importantly, it is safe and highly feasible. Once it is approved for the treatment for AKI, this technique would benefit millions of people who develop AKI. Here, we have shown the efficacy of the VNS after injury in a cisplatin-induced nephropathy model.

Nephrotoxicity appears in around 20–30% of patients after cisplatin administration^20^. It is caused by the accumulation of cisplatin inside the tubular epithelial cells followed by cellular damage, including DNA and mitochondrial damage, and ER stress^21^. In addition to direct tubular injury, inflammation plays an important role in the pathogenesis of cisplatin-induced nephropathy^22^. Following the initial kidney insult, renal parenchymal cells produce several cytokines and chemokines, promoting chemotaxis of inflammatory cells such as neutrophils, macrophages, and T cells. One to two days after cisplatin administration, the numbers of infiltrating neutrophils and macrophages are elevated^23,24^. Considering the timing of VNS application, we inferred that VNS after injury, in contrast to VNS before injury, exerts a renoprotective effect by inhibiting further leukocyte infiltration into the kidney.

CAP is composed of both afferent and efferent arms and both afferent and efferent vagus nerves play important roles. Although its precise mechanism is still to be elucidated, it is estimated that the efferent vagus nerve synapses with the splenic nerve^25^. The activated splenic nerve stimulates CD4 + CD25- T cells (non-regulatory T cells) in the spleen via activation of β2-adrenanergic receptors on those cells^26^. A subset of CD4 + T cells (CD4 + CD44 high CD62L low memory T cells) in the spleen possess the acetylcholine biosynthetic enzyme, choline acetyltransferase (ChAT), and can synthesize acetylcholine^27^, and subsequently, those T cells are supposed to interact with α7 nicotinic acetylcholine receptors on splenic or peritoneal macrophages^28^. The production of pro-inflammatory cytokines, such as TNF-α and interleukins, is suppressed in these activated macrophages. Inoue, for the first time, documented the importance of the peritoneal macrophages in activation of the CAP *in vivo*^28^. Even though the exact relationship between each type of immune cell is not fully understood, the spleen serves as an important place for immune cell interaction and a reserve for immune cells.

As we expected, VNS after cisplatin injection significantly improved and attenuated the extent of tubular injury (Fig. 1). Moreover, this protective effect was completely abolished by splenectomy similar to the IRI-induced tubular injury (Fig. 2)^9^. This is consistent with the previous report demonstrating the importance of the spleen in the CAP^29^.

Adoptive transfer of GTS-21-treated macrophages further reinforced the importance of splenic macrophages in the CAP (Fig. 3). The number of transferred macrophages was relatively small, but their anti-inflammatory effect was potent enough to suppress kidney dysfunction and tubular injury. Thus, we speculate that these cells interact with other immune cells and hinder the proinflammatory process.

Multiple cytokine assay revealed that VNS decreased several chemokines and cytokines related to leukocyte infiltration, supporting our hypothesis (Fig. 4). In IRI or unilateral ureteral obstruction (UUO) mice, G-CSF is upregulated and the number of neutrophils increases in the injured kidney^30^. IL-12 and IL-33 work together as a stimulant to invariant natural killer T cells in the kidney and promote neutrophils and monocytes/macrophage infiltration into the organ^31^. Another study also reported that IL-12 deficient mice had better renal outcomes in an IRI-induced AKI model^32^.

CCL2 (MCP-1) is one of the most potent chemokines, attracting monocytes and macrophages^33^. Circulating CCL2 first recruits monocytes from the bone marrow, and those monocytes are recruited by locally produced CCL2 into the inflamed organs. Following this, local CCL2 induces differentiation and cytokine production of monocytes/macrophages in the kidney. Its pathogenic role has been reported in various kidney diseases, such as membranous nephropathy, SLE, diabetic nephropathy, and autosomal polycystic kidney disease (ADPKD)^33,34^.

Among these cytokines and chemokines, which were suppressed in both plasma and the kidney (Figs. 4,5), we focused on CCL2, since it recruits macrophages, one of the main role players in the CAP. Knocking out CCL2 in the renal tubular cells decreases macrophage infiltration into the kidney and ameliorates cyst formation in ADPKD^34^. In a diabetic nephropathy model, blockade of CCL2/CCR2 signaling using a CCR2 antagonist significantly reduced renal macrophage infiltration and attenuated histological changes caused by diabetes^35^. The pathogenic role of macrophages in AKI has been well established in an IRI mouse model^36^. In the first 48 hours after injury, inducible NO synthase (iNOS)-expressing pro-inflammatory M1 phenotype macrophages predominate in the kidney, whereas in the later phase, arginase-1 (Arg-1)-positive anti-inflammatory macrophages dominate. Reduction of macrophage infiltration, and attenuated pathological changes, and kidney dysfunction are correlated in cisplatin-induced nephropathy mouse models^37,38^.

In the field of rheumatoid arthritis, the relationship between activation of the CAP and attenuation of arthritis via suppression of macrophage infiltration has been reported^39^. GTS-21, a selective agonist for the α7nAChR, effectively decreased the expression level of CCL2 in cisplatin-treated macrophages (data not shown). This supports our *in vivo* data that showed that VNS reduces macrophage infiltration into the kidney via suppression of CCL2 (Fig. 5a). Taken together, these findings suggest that VNS inhibits the pro-inflammatory macrophages into the kidney and ameliorates tubular injury and kidney dysfunction.

Our study documents for the first time the link between macrophage infiltration in the kidney and CAP stimulation, possibly through the modulation of CCL2 expression (Figs. 4–6). Activation of the CAP inhibits NF-κb^40^ and the JAK2/STAT3^41^ pathway, which are major transcriptional pathways for CCL2. In IRI models, VNS pretreatment did not affect the number of macrophages in the kidney^9^, and this discrepancy arises from differences in kidney injury models or differences in the timing of VNS application. Future studies will be needed to clarify the complex molecular mechanisms underlying the relationship between CAP activation and macrophage migration.

In conclusion, we have demonstrated for the first time that VNS after injury protects the kidney from cisplatin-induced nephropathy via CAP activation and prohibition of inflammatory cell infiltration into the kidney. VNS is safe and has potential as a potent therapeutic tool for cisplatin-induced nephropathy. Future studies are needed to show the effectiveness of VNS for other types of AKI.

## Methods

### Animals

C57BL/6 male mice (7–10 weeks old, 20–25 g) were purchased from Sankyo Labo Service Corporation (Tokyo, Japan). All procedures were performed in accordance with the NIH Guide for the Care and Use of Laboratory Animals or the equivalent, and the procedures were approved by the Ethics Committee for Animal Care and Use of The University of Tokyo, Tokyo, Japan.

All surgeries and euthanasia were performed under general anesthesia (medetomidine 0.3 mg/kg, butorphanol 5 mg/kg, and midazolam 4 mg/kg). Cisplatin (25 mg/kg) dissolved in 0.9% normal saline was administered by intraperitoneal injection, and the mice were euthanized 72 hours later under anesthesia by cervical dislocation. The vehicle-treated group received the same volume of normal saline intraperitoneally.

### Vagus nerve stimulation

The left cervical vagus nerve was stimulated as previously reported^9^ using an Isostim Stimulator (A320RC; World Precision Instruments, Sarasota, FL, USA). We isolated the left vagus nerve by mid-cervical incision and placed bipolar silver electrodes (AS633; Cooner Wire). Electrical stimulation (frequency, 5 Hz, square wave; 50 μA; duration, 1 ms) was applied for 10 minutes to the VNS group. For the sham operation, we simply exposed the vagus nerve using an identical incision. After the operation, the anesthesia was reversed with the α2-adrenergic receptor antagonist, atipamezole (0.5 mg/kg). VNS or sham operation were applied to mice 24 hours after cisplatin injection, and 48 hours after VNS or sham operation, kidney functions were evaluated.

### Splenectomy

Five days prior to cisplatin injection, splenectomy was conducted under general anesthesia. The splenic arteries and veins were ligated, and the spleen was removed through a small left back incision. In sham-operated mice, the spleen was just exposed.

### Adoptive transfer of macrophages

Spleens were collected from wild-type donor mice and strained to single-cell suspensions through 40 µm filters with sterile PBS. 1 × 10^8^ cells were labeled with anti-F4/80 MicroBeads (Miltenyi Biotec, Bergisch Gladbach, Germany), and F4/80 positive splenocytes were selected using the magnetic cell separation method (MACS). The cells were incubated with either 100 µM GTS-21 or vehicle for 1 hour and were washed twice with PBS. An injection of 1 × 10^5^ macrophages diluted with 200 µL of normal saline was administered intravenously.

### Evaluation of kidney function

Blood samples were centrifuged at 7,000 g for 5 minutes and plasma was collected. Plasma creatinine level was measured with an enzymatic method based on the manufacturer’s protocol (Wako Pure Chemical Industries, Saitama, Japan). Plasma urea nitrogen level was measured by SRL Inc. (Osaka, Japan).

### Immunohistochemistry

Whole kidneys were cut horizontally into four parts, and one of the central parts was fixed in Mildform 10 N (Wako Pure Chemical Industries) before being embedded in paraffin. Tissue sections (3 μm) were stained with periodic acid-Schiff for evaluation of tubular damage. The tubulointerstitial injury score was graded (0–4) blindly. Semiquantitative scores of tubular injury were graded based on the proportion of injured tubules as follows: none (0); <25% (1); 25%–50% (2); 50%–75% (3); and>75%^4^. Four fields in the outer medulla were selected randomly for each sample, and the average score was calculated.

Expression of Kim-1, which is known to be upregulated early before the elevation of plasma creatinine in cisplatin-induced nephropathy^42^, was assessed by immunostaining using Mouse TIM-1/KIM-1/HAVCR Antibody #AF1817 (R&D Systems, Inc., Minneapolis, MN, USA, diluted 1:200). Embedded tissue was first deparaffinized in Histo-clear (Cosmo Bio, Tokyo, Japan) and rehydrated with ethanol. Sections were boiled in sodium citrate (10 mM, pH 6.0) for 10 minutes using a microwave oven. The sections were blocked with 3% H_2_O_2_ for 15 minutes and Protein Block (Agilent Technologies, Inc., Santa Clara, USA) for 10 min at room temperature. A VECTASTAIN ABC-HRP Kit, Peroxidase (Goat-IgG) - (PK-4005) (Vector Laboratories, Burlingame, CA, USA) were used for the following steps. The signal was detected using ImmPACT DAB Substrate, Peroxidase (HRP) - (SK-4105) (Vector Laboratories). The percentage of positive signal area was analyzed using ImageJ software (National Institutes of Health, Bethesda, MD, USA).

For immunostaining of F4/80 positive macrophages, kidneys were fixed in methyl Carnoy’s solution (60% methanol, 30% chloroform, and 10% acetic acid). Renal macrophages were stained using rat anti-mouse F4/80 antibody MCA497 (Cl: A3-1) (Bio-Rad Laboratories, Hercules, CA, USA; diluted 1:400) and subsequent incubation with Histofine Simple Stain Mouse MAX-PO (Rat) (Nichirei Biosciences Inc., Tokyo, Japan). The number of infiltrating macrophages was counted as the mean value of three randomly selected areas.

### RNA isolation and quantitative real-time polymerase chain reaction (PCR)

Renal mRNA was isolated from the edge of one of the slices from the left kidneys using RNAiso Plus (Takara Bio Inc., Shiga, Japan). For RNA isolation from cells, we used a RNeasy Mini Kit (QIAGEN, Venlo, Netherlands). Reverse transcription was performed using PrimeScript RT master mix (Takara Bio). The cDNA was then used to determine relative mRNA expression with Fast SYBR Green Master Mix (Applied Biosystems, Waltham, MA, USA) on a StepOnePlus Real-Time PCR System (Applied Biosystems). Relative expression was calculated using the comparative cycle threshold (CT; 2 − ΔΔCt) method. Primer sequences are listed in Table 1.

**Table 1:** Primer list.

Kim-1 Fw	mouse	ACATATCGTGGAATCACAACGAC
Kim-1 Rv	mouse	ACTGCTCTTCTGATAGGTGACA
TNFα Fw	mouse	GCCTCTTCTCATTCCTGCTTG
TNFα Rv	mouse	CTGATGAGAGGGAGGCCATT
GAPDH Fw	mouse	AGGTCGGTGTGAACGGATTTG
GAPDH Rv	mouse	TGTAGACCATGTAGTTGAGGTCA
CCL11 Fw	mouse	GAATCACCAACAACAGATGCAC
CCL11 Rv	mouse	ATCCTGGACCCACTTCTTCTT
G-CSF Fw	mouse	CAGCCCAGATCACCCAGAATC
G-CSF Rv	mouse	GCTGCAGGGCCATTAGCTTC
IL-12b Fw	mouse	ATTACTCCGGACGGTTCACG
IL-12b Rv	mouse	ACGCCATTCCACATGTCACT
CCL2 Fw	mouse	GACCTTAGGGCAGATGCAGT
CCL2 Rv	mouse	AGCTGTAGTTTTTGTCACCAAGC

### Cytokine/chemokine immunoassay of plasma

A Bio-Plex Pro Mouse Cytokine GI 23-plex panel was used to determine the plasma cytokine and chemokine levels in the cisplatin-induced nephropathy mouse model. The assay was performed based on the Bio-Plex Pro assay protocol (Bio-Rad). Clustering was performed using Cluster 3.0^43^, and a heatmap was created with Java TreeView 1.1 6r4^44^.

### Flow cytometry analysis

Kidney suspensions were prepared from mice injected with cisplatin with or without VNS. Kidneys were weighed, minced, and incubated with collagenase (Sigma-Aldrich, St Louis, MO, USA) and DNase I (Sigma-Aldrich) in RPMI buffer with 10% FBS for 40 minutes at 37°C. The digested kidney tissue suspension was filtered through a 70-μm and 40-μm cell strainer (Greiner Bio-One, Kremsmünster, Austria) via the rubber end of a 2.5-ml syringe plunger and then centrifuged at 500 g for 5 minutes at 4°C. The cells were centrifuged again, the supernatant was discarded, and the cells were resuspended with Flow Cytometry Staining Buffer (Thermo Fisher Scientific, Santa Clara, CA, USA). After blocking nonspecific Fc binding with anti-mouse CD16/32 (2.4G2; Thermo Fisher Scientific), fresh kidney suspensions were incubated with the following antibodies: anti-mouse CD45-APC-eFluor 780 (30-F11; Thermo Fisher Scientific), CD11b-eFluor 450 (M1/70; Thermo Fisher Scientific), CD3-Alexa Fluor 700 (17A2; Thermo Fisher Scientific), Ly6G-APC (1A8; Thermo Fisher Scientific), MHC class II-FITC (NIMR-4; Thermo Fisher Scientific), CD11c-FITC (N418; Thermo Fisher Scientific), F4/80-PE (BM8; Thermo Fisher Scientific), B220-PE Cy7 (RA3-6B2; Thermo Fisher Scientific) and 7-AAD (Thermo Fisher Scientific) was used to exclude dead cells. Counting Beads (CountBright Absolute Counting Beads, Thermo Fisher Scientific) were used to calculate the cell number (g^−1^ kidney) as follows: CD45 cell absolute count (g^−1^ kidney) = (events of CD45 cells counted/total number of beads counted × input bead number)/g kidney. The leukocyte subset cell number (g^−1^ kidney) was multiplied by the CD45 cell number and by the percentage of the subset. For compensation, compensation beads (UltraComp eBeads; Thermo Fisher Scientific) were used. Flow cytometry data were acquired on an Attune NxT Flow Cytometer (A24860; Thermo Fisher Scientific) and analyzed by FlowJo software 10.6 (BD, Franklin Lakes, NJ, USA). The same gating strategy as previously reported was applied in this study^9^.

### Statistics

All data are expressed as mean ± standard error of the mean (SEM) with individual values in dot plots. Data were analyzed using a two-way ANOVA for multiple comparisons or a Student’s t-test for comparison between two groups. A P-value of *P* < 0.05 was defined as a significant difference. All the analyses were performed with GraphPad Prism version 8.3 (GraphPad Software, San Diego, CA, USA).

## Supplementary information

Supplementary information.

Supplementary information2.

## Data Availability

No datasets were generated or analyzed during the current study.
