# Supplementary material for: Vagus nerve stimulation even after injury ameliorates cisplatin-induced nephropathy via reducing macrophage infiltration
Source: Sci Rep. 2020 Jun 11;10:9472. doi: 10.1038/s41598-020-66295-0 (PMC7290038; doi:10.1038/s41598-020-66295-0)
Supplement: Supplementary file 1 — Supplementary information. [file 41598_2020_66295_MOESM1_ESM.pdf]

Vagus nerve stimulation even after injury ameliorates cisplatin-induced nephropathy via reducing macrophage infiltration

Rie Uni[1,2], Tsuyoshi Inoue[2*], Yasuna Nakamura[1], Daichi Fukaya[2], Sho Hasegawa[1,2], Chia-Hsien Wu[1], Rie Fujii[1,2], Bongkod Surattichaiyakul[2], Wachirasek Peerapanyasut[3], Atsuko Ozeki[4], Nobuyoshi Akimitsu[4], Youichiro Wada[4], Masaomi Nangaku[1], Reiko Inagi[2]

1 Division of Nephrology and Endocrinology, The University of Tokyo Graduate School of Medicine, Bunkyo-ku, Tokyo, 113-8655, Japan, 2 Division of CKD Pathophysiology, The University of Tokyo Graduate School of Medicine, Bunkyo-ku, Tokyo, 113-8655, Japan, 3 Mahidol University, Nakhonsawan Campus, Nakhonsawan, 60130, Thailand, 4 Isotope Science Center, The University of Tokyo, Bunkyo-ku, Tokyo, 113-0032, Japan

*Corresponding author

Corresponding to tsinoue-tky@umin.ac.jp

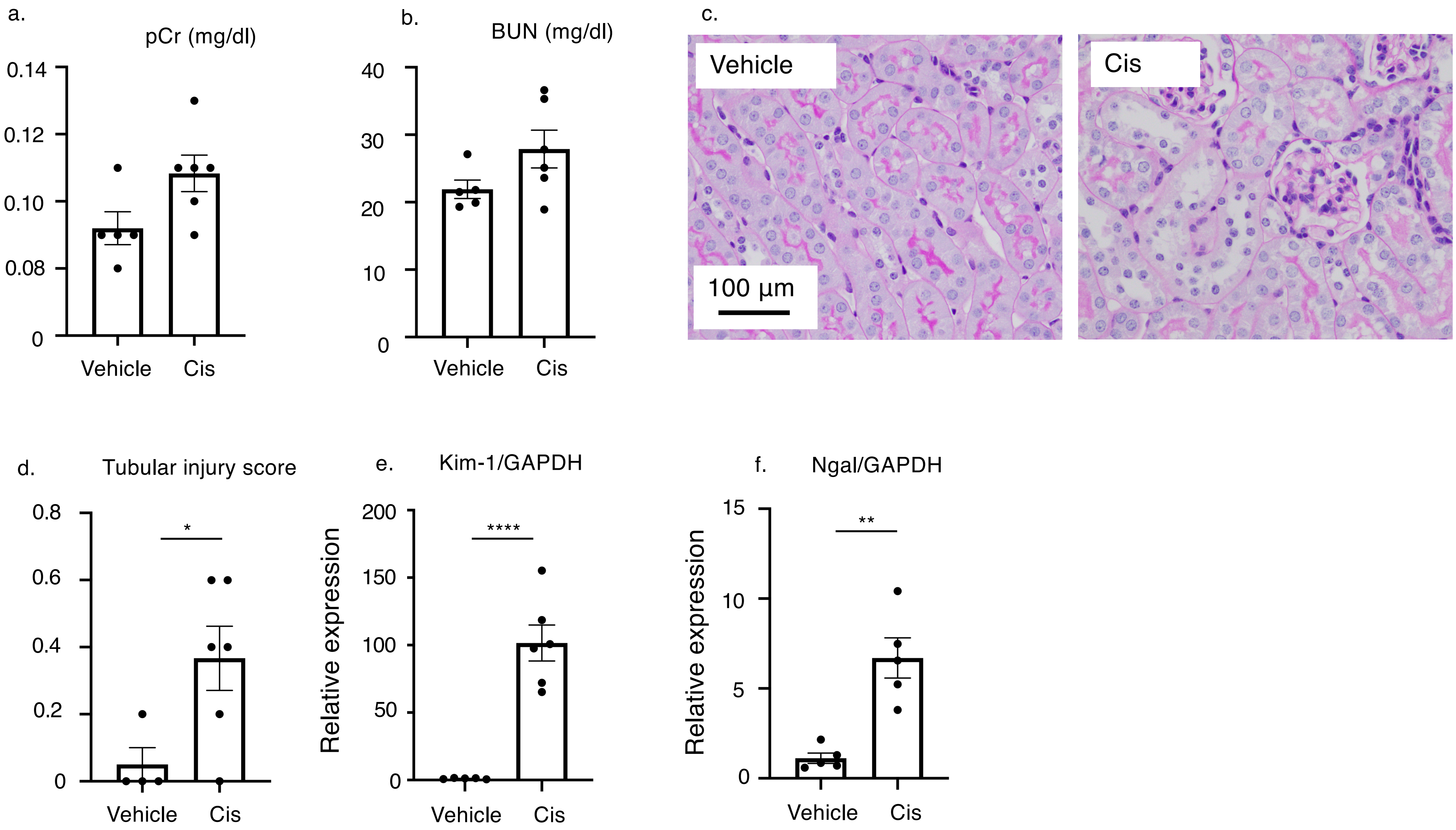

a.
pCr (mg/dl)
0.14
0.12
0.10
0.08
0
Vehicle
Cis
b.
BUN (mg/dl)
40
30
20
10
0
Vehicle
Cis
c.
Vehicle
Cis
100 µm
d.
Tubular injury score
0.8
0.6
0.4
0.2
0
*
Vehicle
Cis
e.
Kim-1/GAPDH
Relative expression
200
150
100
50
0
****
Vehicle
Cis
f.
Ngal/GAPDH
Relative expression
15
10
5
0
**
Vehicle
Cis

Supplementary Figure 1

Kidney injury is already established 24 hours after cisplatin injection.

(a, b) Plasma creatinine and BUN levels were elevated 24 hours after cisplatin injection, but the increases were not statistically significant (plasma creatinine: 0.09 ± 0.00 and 0.11 ± 0.01 mg/dl, *P*=0.056; BUN: 21.9 ± 1.2 and 27.9 ± 2.6 mg/dl, *P*=0.11; for vehicle and cisplatin group, respectively; n=4 or 5). (c, d) Representative pictures of PAS staining and tubular injury score. Cisplatin caused tubular injury only 24 hours after its administration (tubular injury score: 0.05 ± 0.04 and 0.37 ± 0.10 for vehicle group and cisplatin group, respectively; *P*=0.036). (e, f) The expression levels of the early kidney injury markers Kim-1 and Ngal mRNAs in the kidney were also increased (relative expression of Kim-1: 1.10 ± 0.19 and 101.6 ± 12.2, *P*<0.0001; relative expression of Ngal: 1.12 ± 0.25 and 6.67 ± 1.00, *P*=0.0013; for vehicle group and cisplatin group, respectively; n=5 or 6).

Data are expressed as mean ± SEM and analyzed using a Student's t test.

*P < 0.05, **P < 0.01, **** P<0.0001

Cis, cisplatin; pCr, plasma creatinine; BUN, blood urea nitrogen; SEM, standard error of the mean

Scale bar, 100 µm.

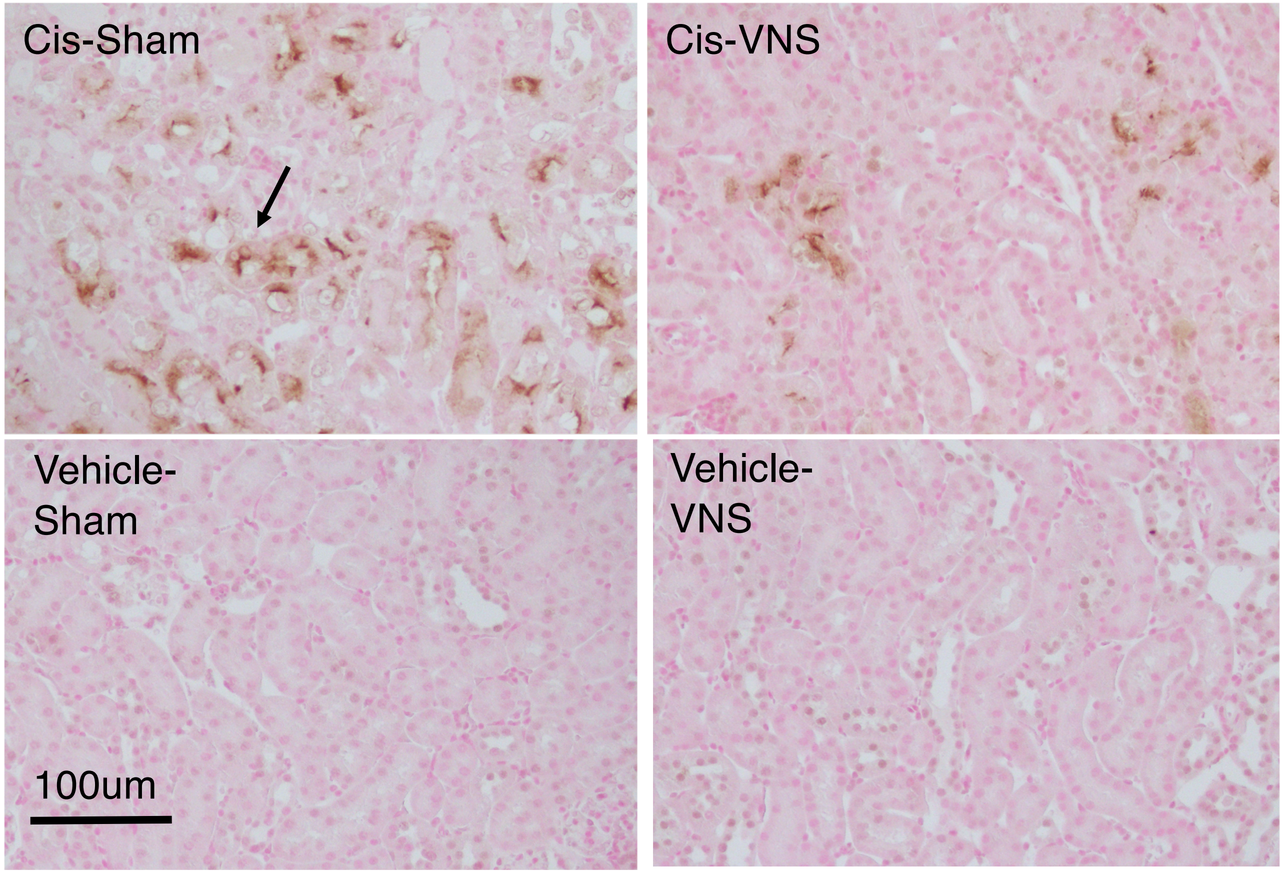
Cis-Sham
Cis-VNS
Vehicle-
Sham
Vehicle-
VNS
100um

Supplementary Figure 2

High power field view of Kim-1 immunohistochemistry (Figure 1d).

VNS was applied 24 hours after intraperitoneal single injection of cisplatin (25 mg/kg).

Black arrow indicates Kim-1-positive area stained with brown.

Cis, cisplatin; VNS, Vagus nerve stimulation

Scale bar, 100 μm.
