# Supplementary material for: Vagus nerve stimulation even after injury ameliorates cisplatin-induced nephropathy via reducing macrophage infiltration
Source: Sci Rep. 2020 Jun 11;10:9472. doi: 10.1038/s41598-020-66295-0 (PMC7290038; doi:10.1038/s41598-020-66295-0)
Supplement: Supplementary file 2 — Supplementary information2. [file 41598_2020_66295_MOESM2_ESM.pdf]

Vagus nerve stimulation even after injury ameliorates cisplatin-induced nephropathy via reducing macrophage infiltration

Rie Uni[1,2], Tsuyoshi Inoue[2*], Yasuna Nakamura[1], Daichi Fukaya[2], Sho Hasegawa[1,2], Chia-Hsien Wu[1], Rie Fujii[1,2], Bongkod Surattichaiyakul[2], Wachirasek Peerapanyasut[3], Atsuko Ozeki[4], Nobuyoshi Akimitsu[4], Youichiro Wada[4], Masaomi Nangaku[1], Reiko Inagi[2]

1 Division of Nephrology and Endocrinology, The University of Tokyo Graduate School of Medicine, Bunkyo-ku, Tokyo, 113-8655, Japan, 2 Division of CKD Pathophysiology, The University of Tokyo Graduate School of Medicine, Bunkyo-ku, Tokyo, 113-8655, Japan, 3 Mahidol University, Nakhonsawan Campus, Nakhonsawan, 60130, Thailand, 4 Isotope Science Center, The University of Tokyo, Bunkyo-ku, Tokyo, 113-0032, Japan

*Corresponding author

Corresponding to tsinoue-tky@umin.ac.jp

Supplementary Table 1

Various inflammatory cytokine expression levels in plasma (mean, pg/ml).

	Vehicle-Sham	(SD)	Vehicle-VNS	(SD)	Cis-Sham	(SD)	Cis-VNS	(SD)
IL-1α	20.3	6.6	43.7	25.1	19.4	17.2	15.3	16.5
CCL11	715.8	247.8	843.0	167.6	2565.5	787.0	1559.1*	397.6
G-CSF	402.9	97.5	351.5	254.0	1010.4	551.9	355.4*	239.4
IL-12(p40)	529.7	148.2	355.6	90.8	1154.1	350.6	723.1*	305.8
IL-10	34.6	14.0	16.1	17.3	71.5	34.9	64.8	34.4
IL-6	13.3	5.2	6.8	5.0	23.0	10.2	15.2	10.8
CCL2	238.3	54.2	104.5	52.2	368.9	128.1	282.0*	131.9
IL-2	9.0	1.8	4.0	2.1	11.3	5.1	9.1	4.5
IL-5	19.5	6.0	10.2	6.7	25.3	13.3	17.2	7.1
RANTES	107.7	21.5	78.9	44.1	127.9	31.2	102.6	43.3
KC	202.2	33.8	106.7	51.4	169.8	46.7	191.1	54.4
IL-9	58.2	12.2	33.8	20.1	34.8	19.3	65.0	68.9
MIP-1β	156.8	23.4	110.0	43.4	128.2	31.0	146.2	39.9
IL-13	229.1	44.9	124.3	64.9	158.2	68.9	195.4	151.5
MIP-1α	11.6	2.1	6.9	3.4	6.9	2.6	8.8	3.3
IL-17	27.2	7.5	13.9	8.9	15.0	6.7	19.8	7.8
GM-CSF	94.3	21.2	55.6	34.2	57.8	22.4	76.4	23.9
IL-4	9.5	2.1	4.5	2.9	4.9	2.6	7.1	3.9
IL-3	6.8	2.2	3.5	1.6	3.6	1.9	5.2	2.6
IL-1β	45.0	10.0	19.8	13.1	23.7	13.0	30.2	13.8
TNF-α	156.7	36.0	68.7	50.1	81.8	41.4	111.8	58.1
IFN-γ	95.1	18.7	57.5	33.2	48.4	30.5	73.9	31.9
IL-12(p70)	461.9	113.1	411.5	375.5	231.6	125.0	353.5	147.3

*Decreased by VNS significantly compared to Cis-sham group. (CCL11: $P<0.0001$, G-CSF: $P=0.0002$, IL-12(p40): $P=0.0009$, CCL2: $P=0.0325$) (two-way analysis of variance followed by Sidak post-hoc

test)

VNS, vagus nerve stimulation; Cis, cisplatin; SD, standard deviation
